# Complete Genome Sequences of Porcine Parvovirus 2 Isolated from Swine in the Republic of Korea

**DOI:** 10.1128/genomeA.01738-16

**Published:** 2017-04-13

**Authors:** Ju-Yeon Lee, Eun-ju Kim, In-soo Cho, Kyoung-Ki Lee, Yeun-Kyung Shin

**Affiliations:** a Viral Disease Division, Animal and Plant Quarantine Agency, Gimcheon, Republic of Korea; b Animal Disease Diagnostic Division, Animal and Plant Quarantine Agency, Gimcheon, Republic of Korea

## Abstract

Here, we report sequences of the porcine parvovirus 2 (PPV2) genome, isolated from pigs in the Republic of Korea in 2016. The sequences of open reading frames 1 and 2 (ORF1 and ORF2) had a 98.8 to 98.9% homology with the US135 strain and 98.8% homology with the US523 strain, respectively. This is the first study to report the PPV2 genome in South Korean pigs.

## GENOME ANNOUNCEMENT

Parvoviruses are small nonenveloped viruses with single-stranded, linear DNA genomes of approximately 5 to 6 kb. The *Parvoviridae* family is divided into two subfamilies: *Densovirinae* and *Parvovirinae*. The subfamily *Parvovirinae* is subdivided into eight genera: *Amdoparvovirus*, *Aveparvovirus*, *Bocaparvovirus*, *Copiparvovirus*, *Dependoparvovirus*, *Erythroparvovirus*, *Protoparvovirus*, and *Tetraparvovirus* (1). Porcine parvovirus 2 (PPV2), a member of the *Tetraparvovirus* genus, was first detected in Myanmar in 2001 (2) and also reported in several countries worldwide such as China (3), Hungary (4), the United States (5), Germany (6), Thailand (7), and Japan (8).

Here, we report two complete sequences of PPV2 genomes which were identified in lung tissues of fattening pigs from a slaughter house located in the Gyeongbuk province in the Republic of Korea in 2016. DNAs were extracted from 10% lung homogenized samples with a DNA extraction kit (Intron, Inc., Republic of Korea). PCR amplifications were performed for a total of 15 of the lung tissues with a pair of primers (PPV2-DF and PPV2-DR) that detected open reading frame 2 (ORF2) (5). PPV2 was detected in 13 of the 15 lung tissues.

For whole-genome sequencing, ORF1 and ORF2 were amplified with two pairs of primers (PPV2-F6 and PPV2-2164-AR and PPV2-AF and PPV2-AR) to 2,100 bp (5) and 3,427 bp (9), respectively. The amplicons were purified with an agarose gel extraction kit (Qiagen, Germany) and ligated into the pDrive vector (Qiagen) following the manufacturer’s instructions. The ligation product was transformed into *Escherichia coli* DH5α–competent cells and then incubated at 37°C overnight. For plasmid DNA extraction, a plasmid miniprep kit (Inclone, Republic of Korea) was used following the manufacturer’s instructions. The PPV2 sequences in the plasmids were confirmed by DNA sequencing (Macrogen, Republic of Korea).

The sequences of PPV2 were analyzed with DNAstar version 5.0 and two complete PPV2 ORF1 (1,986 nucleotides [nt]) and ORF2 (2,936 nt) sequences were acquired (5,350 bp). The two sequences (GBGW1 and GBGW2) showed a nucleotide identity of 99.8% in ORF1 and 99.9% in ORF2.

Phylogenetic analysis performed with MEGA version 6.0 (10) showed that the South Korean PPV2s were closely related to PPV2 US135 and US523, which were isolated in 2011 in the United States (GenBank accession no. JX101461 and JX101462). The sequences of ORF1 and ORF2 had a 98.8 to 98.9% homology with the US135 strain and 98.8% with the US523 strain, respectively.

This is the first study to report the PPV2 genome in South Korean swine. It is still not known whether PPV2 is related to a clinical disease (8). An association of PPV2 with porcine circovirus 2 and porcine circovirus–associated disease was identified, supporting further diagnostic investigations and research based on this pathogenesis (11). To better understand PPV2, the virus will need to be isolated.

**Accession number(s).** The genome sequences of PPV2 ORF1 and ORF2 were deposited in GenBank under the accession numbers KY018935 (GBGW1) and KY018936 (GBGW2), respectively.
